# Effects of enhanced external counterpulsation on exercise capacity and quality of life in patients with chronic heart failure: A meta-analysis

**DOI:** 10.1097/MD.0000000000026536

**Published:** 2021-07-09

**Authors:** Zhao-Feng Zhou, Da-jie Wang, Xu-Mei Li, Cheng-Lin Zhang, Chun-Yang Wu

**Affiliations:** The Affiliated Yancheng Hospital, School of Medicine, Southeast University, Yancheng, Jiangsu Province, China.

**Keywords:** enhanced external counterpulsation, heart failure, meta-analysis

## Abstract

**Background:**

This meta-analysis aimed to synthesize randomized controlled trials to evaluate the effects of enhanced external counterpulsation (EECP) on exercise capacity and quality of life in patients with chronic heart failure (CHF).

**Methods:**

Both English and Chinese databases were searched from their inception to June 30, 2020 (PubMed, EMBASE, Cochrane Library, CINAHL (EBSCO), Web of Science for English publications and Chinese Biomedical Database, China National Knowledge Infrastructure, Wanfang Data for Chinese publication). Titles, abstracts, and full-text articles were screened against study inclusion criteria: randomized controlled trials studying EECP intervention for patients with CHF. The meta-analysis was conducted with Revman 5.3 or STATA 16.0.

**Results:**

Eight randomized controlled trials were included. EECP induced significant improvement in 6-min walking distance (WMD=84.79 m; 95% CI, 47.64 to 121.95; *P* < .00001). Moreover, EECP was beneficial for left ventricular ejection fraction (SMD = 0.64; 95% CI,0.29 to 1.00; *P* = .0004), and N-terminal pro brain natriuretic peptide (SMD = −0.61; 95%CI, −1.20 to −0.01; *P* = 0.04).However, compared with the control groups, EECP did not significantly reduce the Minnesota Living with Heart Failure Questionnaire scores(WMD, −9.28; 95% CI, −19.30 to 0.75; *P* = 0.07).

**Conclusions:**

Despite heterogeneity and risk of bias, this meta-analysis confirms that EECP can improve exercise capacity in CHF patients, especially the elderly. However, the evidence that EECP improves the quality of life in patients with CHF is still insufficient. More and larger well-designed randomized controlled trials are still warranted.

**Registration information:**

PROSPERO registration no. CRD 42020188848.

## 1 Introduction

Heart failure (HF) is a serious clinical and public health problem, due to any structural or functional damage of ventricular filling or blood ejection. As a result of global population growth and aging, heart failure remains a rising global epidemic with an estimated prevalence of over 64.3 million people worldwide.^[1]^ The prevalence of HF varies between 0.1% and 6.7% worldwide.^[2]^ HF is one of the leading causes of hospitalization and readmission, which is responsible for a massive economic burden on our healthcare systems. More than 1 million people in the USA are hospitalized each year for heart failure, and the 1-year readmission rate is higher than 25%.^[3]^ Although great progress has been made in the treatment of heart failure in the past few decades, especially in medical and device therapy, the hospitalization rate and mortality rate of patients with HF are still very high. Therefore, cardiac rehabilitation has become more and more prevalent in HF treatment. As an important means of cardiac rehabilitation, enhanced external counterpulsation (EECP) has been paid more and more attention, especially in some elderly patients.^[4,5]^

Several trials have demonstrated that EECP, as a noninvasive therapy, can improve the symptoms, cardiac function, exercise tolerance, quality of life (QOL) in patients with HF, and reduce the readmission rate.^[6,7]^ However, Taguchi et al. reported that the hemodynamic effect of EECP may lead to a sharp increase in right atrial mean pressure and pulmonary capillary wedge pressure in patients with HF accompanied by left ventricular dysfunction, which may lead to deterioration of the disease.^[8]^ Therefore, in the treatment of HF, the role of EECP is still controversial, and its application needs more evidence-based support. Here, a systematic review and meta-analysis was performed to assess the effects of EECP on exercise capacity and QOL in patients with HF.

## 2 Methods

### 2.1 Search strategy

We will retrieve articles from the following electronic databases: PubMed, EMBASE, Cochrane Library, CINAHL (EBSCO), Web of Science, Chinese Biomedical Database, China National Knowledge Infrastructure, and Wanfang Data. The publication period will be from inception to Sept. 30, 2020. Keywords used in these searches were EECP or external counterpulsation or EECP in combination with heart failure or heart decompensation or myocardial failure or cardiac failure or left ventricular dysfunction or left ventricular systolic dysfunction or reduced left ventricular ejection fraction. No language restrictions. The reference lists of relevant articles were screened and checked to find more eligible studies.

### 2.2 Study selection

Inclusion criteria for studies were applied as follows: 1) Study design: randomized controlled trial, and reported in a complete paper article. 2) Participants: patients were diagnosed with chronic heart failure (CHF) with reduced ejection fraction, or mid-range ejection fraction, or preserved ejection fraction. 3) Intervention group: Patients in the intervention group were implemented with EECP. The standard treatment for EECP is 36 hours (1 h/d, 6 times/wk, 6 wk), or 35 hours (1 h/d, 5 times/wk, 7 weeks). 4) Control group: Patients of the control group were given conventional therapy including dietary, routing nursing, and oral pharmacologic therapy, or only pharmacotherapy, or sham EECP. 5) Primary outcome measures: Use the following indicators to evaluate exercise capacity, including peak VO2, VO2 maximum, exercise time, walking distance (such as [6MWD] 6-minute walking distance), or endurance exercise. SF-36, the Minnesota Living with Heart Failure Questionnaire (MLHFQ), or other validated questionnaires were used to assess the QOL. Other outcomes such as B-type natriuretic peptide or N-terminal pro-brain natriuretic peptide, left ventricular ejection fraction (LVEF), and serious adverse events (SAES) related to EECP were classified as secondary outcomes. No limitations were placed on the race population, religion, or gender.

### 2.3 Data extraction and quality assessment

Data extraction was performed by 2 independent reviewers (Zhaofeng Zhou and Dajie Wang) using predefined criteria. Relevant data extracted for the study design included study characteristics (e.g., author or year or country), participant characteristics (e.g., age or sample size or dropout rate or diagnosis of heart failure etiology), intervention used for the control group, and outcomes measured. Study quality and risk of bias were assessed using the Cochrane Collaboration tool. Any discrepancies on the extracted data or quality assessment between two reviewers were resolved through discussion with the third independent reviewer (Chunyang Wu).

### 2.4 Statistical analysis

RevMan 5.3 and STATA 16.0 were used to perform the meta-analysis. For continuous variable data, changes between the baseline and endpoints were used to assess the intervention or control effects. Main outcomes were expressed as weighted average differences (WMD; if the data were of the same units) or standardized average differences (SMD; if the data were of different units or there was a significant difference) and 95% confidence intervals (CIs). *P* < .05 was considered statistically significant. Heterogeneity was evaluated by Cochran *Q* statistic and quantified by *I*^2^ index. When *P* < .10 and *I*^2^>50% indicated significant heterogeneity, a random-effects model was used; otherwise, a fixed-effect model was used. Tables and narrative methods were used to report other outcomes that could not aggregate in the meta-analysis.

Perform a sensitivity analysis to estimate the impact of a single study on the overall summary results. If possible, subgroup analyses were performed according to different follow-up time or LVEF levels or etiology of CHF. If more than 10 studies were included, funnel plots or Egger rank correlation test, or Egger linear regression test was used to assess potential publication bias.

## 3 Results

### 3.1 Literature search

**Figure 1:**
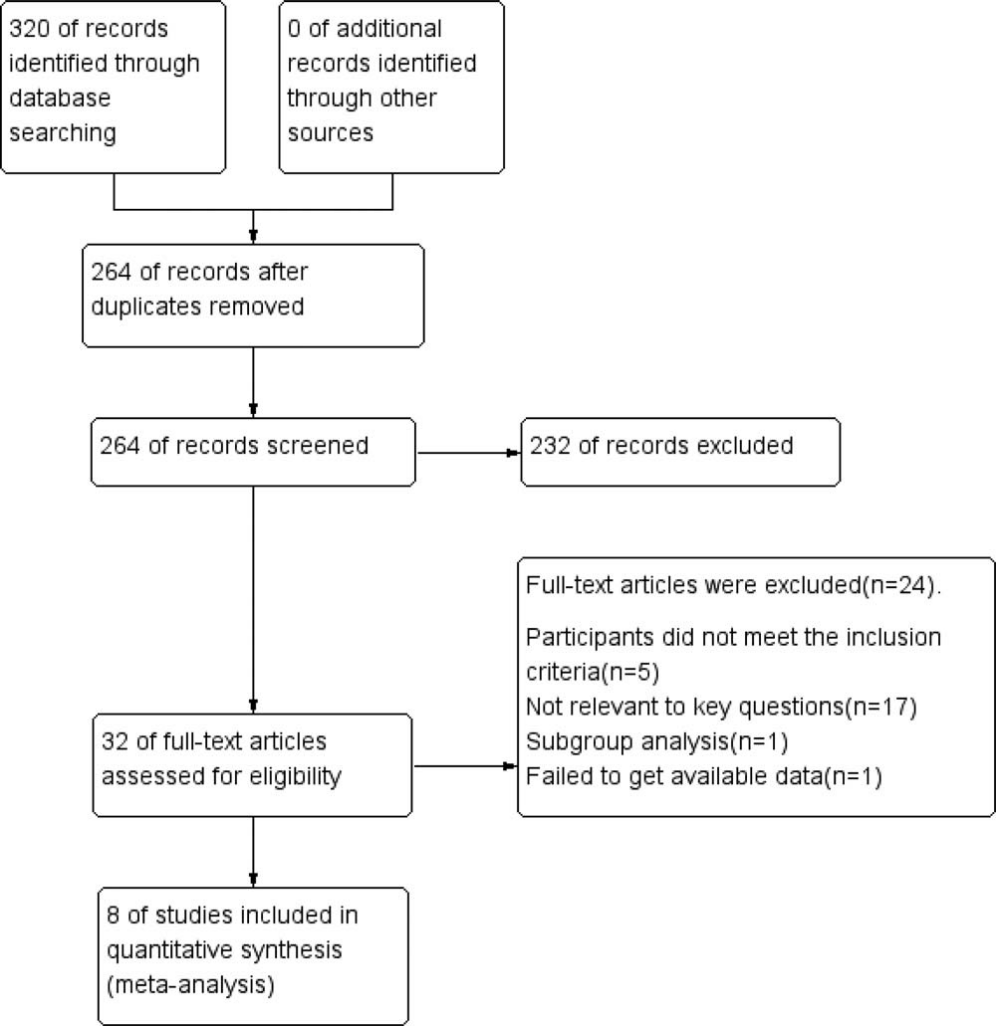
Flow diagram of search and selection of studies.

A total of 320 records were retrieved from the database through our retrieval strategy. After eliminating the duplicate items, 264 records were screened for qualification. Through reading the titles and abstracts, 235 records were excluded because they did not satisfy the inclusion criteria or could not obtain the original data. The remaining 32 full texts were read, 9 randomized controlled trials (RCTs) (6^[9–14]^ in Chinese and 3^[15–17]^ in English) were selected, but 1^[17]^ of them was a subgroup analysis of the congestive heart failure (PEECH) trial.^[15]^ Finally, 8^[9–16]^ RCTs were selected for this report, and meta-analysis (Fig. 1).

### 3.2 Study characteristics

The main characteristics of the included studies are described in Table 1. In these 8 studies, 6^[9–14]^ were carried out in China, 1^[15]^ in the United States, and 1^[16]^ in Indonesia. A total of 823 participants were enrolled in the 8 studies (experimental groups vs control groups, 409 vs 414, respectively), and the sample size per RCT ranged from 40 to 180. The mean age was 64.6 years. Patients of the control group were treated with conventional therapy in 7 studies, and with sham EECP in 1^[16]^ study. The follow-up time ranged from 5 weeks to 6 months.

**Table 1:** Characteristics of RCTs included in the meta-analysis.

	Participant characteristics								
Study	Sample Size (I/C)	Male (%)	Age, Mean (SD or Range)	Etiology of heart failure	NYHA class;LVEF (SD or Range)	Intervention group	Control group	Main outcomes	Serious adverse events related to EECP
Zhang et al, 2019, China	53/53	I:39(73.58);C:38(71.70)	I:66.82 ± 5.61;C:66.02 ± 6.93	NM	II-III; NS	One-hour daily sessions for a total of 35 h in 5 wk	CT	Before and after treatment: LVEF, 6MWD, NT-proBNP	NM
Liao et al, 2018, China	40/40	I:23(57.5);C:21(52.5)	I:68.5 ± 6.4;C:67.2 ± 5.5	NM	NM; 40%-49%	One-h daily sessions for a total of 36 h in 6 wk	CT	Before and 3 mo after treatment: 6MWD, NT-proBNP, MLHFQ	NM
Li et al, 2017, China	30/30	NM	65-80	NM	II-III; <50%	One-h daily sessions for a total of 35 h in 7 wk	CT	Before and 3 mo after treatment:LVEF, 6MWD, NT-proBNP	NM
Liu et al, 2014, China	20/20	I:11(55.0);C:12(60.0)	I:71.8 ± 4.4;C:70.1 ± 4.3	NM	II-III;<50%	One-h daily sessions for a total of 35 h in 7 wk	CT	Before and 3 mo after treatment:6MWD,NT-proBNP	NM
Li et al, 2018, China	53/53	I:34(64.15);C:34(64.15)	I: 67.9 ± 10.9: C:65.7 ± 12.2	IHF	II-III; NS	One-hour daily sessions for a total of 35 h in 5 wk	CT	Before and after treatment:LVEF,6MWD	NM
Yu et al, 2017, China	92/88	I:64(69.6);C:56(63.6)	I:64.82 ± 8.27;C:65.39 ± 7.64	IHF	II-III; NS	One-h daily sessions for a total of 36 h in 6 wk	CT	Before and after treatment:LVEF,NT-proBNP	No
Arthur et al, 2006, United States	71/81	I: 72(77.4);C: 71(75.5)	I:62.4 ± 11.7;C:63.0 ± 10.4	IHF and NIHF	II-III; ≤35%	One-h daily sessions for a total of 35 h in 7 to 8 wk	CT	Before treatment; 1 wk,3 months,and 6 mo after treatment:exercise duration,peak VO2,MLHFQ	3 (worsening heart failure: one patient; Pulmonary embolism:one patient; Deep venous thrombosis:one patient)
Starry et al, 2015, Indonesia	50/49	I:36(72);C:38(77.6)	I:60.54 ± 8.6;C:62.43 ± 12.06	IHF, hypertension heart disease, coronary heart disease or acute coronary syndrome	I-II; NS	One-hour daily sessions for a total of 36 h in 7 wk	CT and sham EECP	Before and after treatment:6WMD	NM

6MWD = 6-minute walking distance, C = control group, CT = conventional therapy, I = intervention group, IHF = ischemic cardiomyopathy, LVEF = left ventricular ejection fraction, MLHFQ = Minnesota Living with Heart Failure Questionnaire, NIHF = non ischemic cardiomyopathy, NM = Not mentioned, NS = not specified, NT pro-BNP = N-terminal pro-brain natriuretic peptide, NYHA = New York Heart Association.

### 3.3 Risk of bias assessment

**Figure 2:**
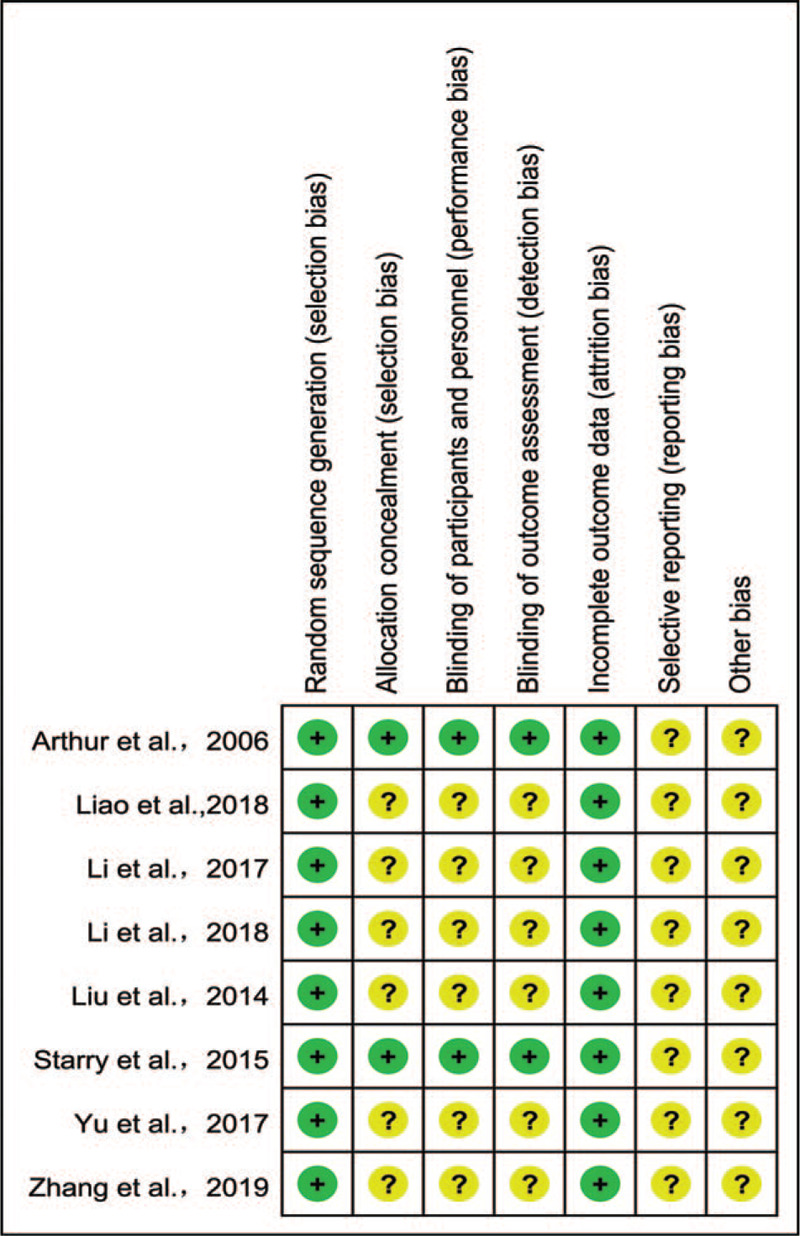
Risk of bias summary: review authors’judgements about each risk of bias item for each included study.

**Figure 3:**
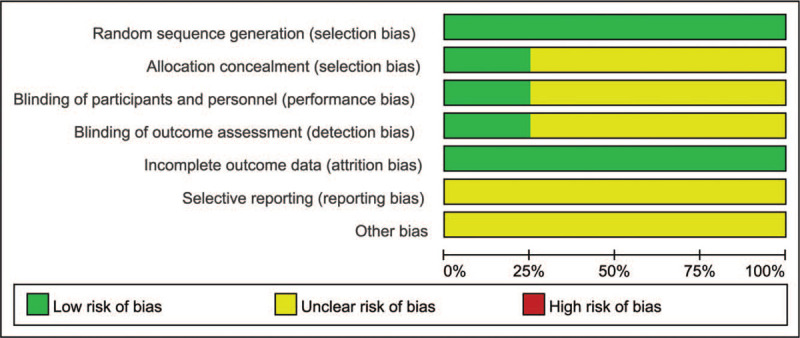
Review judgements regarding each risk of bias item presented as percentages across all included studies.

The bias condition of the selected studies was shown in Figures 2 and 3. We assessed the risk of bias in all included studies. All the 8 included studies mentioned “randomization,” of which 6^[9–13,15]^ used the random number table method, 1^[16]^ used the considered envelope, and 1^[14]^ did not describe it in detail. 2^[15,16]^ of them mentioned the blind method, while the others did not mention the blind method and concealment of allocation scheme. In all trials, the risk of incomplete outcome data was low. Due to the lack of available research protocols, all studies had an unclear risk of bias in selective reporting. Other bias was assessed as unclear because no additional information could be obtained from the original authors.

### 3.4 Primary outcomes

**Figure 4:**
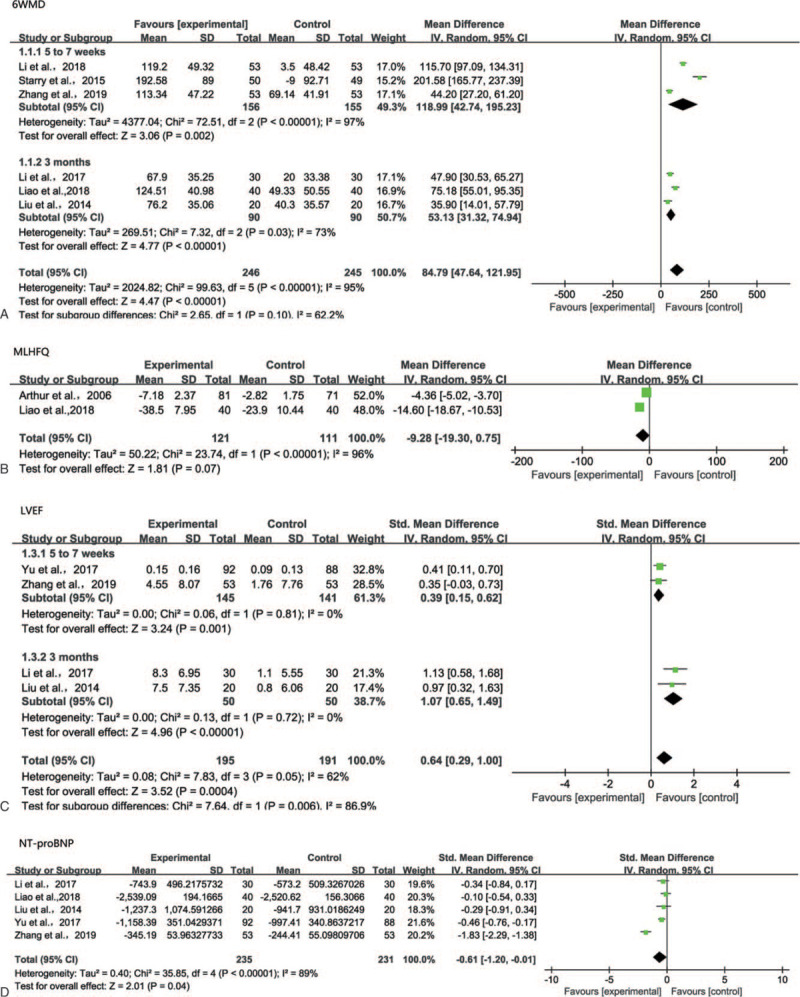
A. A forest plot of the subgroup analyses of 6WMD based on different follow-up time. B. A forest plot for MLHFQ from two research works. C. A forest plot of the subgroup analyses of LVEF based on different follow-up time. D. A forest plot for NT-pro BNP from five research works.

Six RCTs included the results of 6MWD.^[9–13,16]^ Combining these studies, the results suggested EECP was associated with a significantly improved 6MWD(random-effects model: WMD, 84.79 m; 95% CI, 47.64 to 121.95; *P* < .00001; Fig. 4A). The heterogeneity was considerable (*P* < .0001; *I*^2^ = 95%). Then, sensitivity analyses were used to explore potential sources of heterogeneity. We excluded individual studies for sensitivity analyses, and the results showed no obvious differences between the selected studies. According to the subgroup analysis based on different follow-up time, the heterogeneity reduced (Fig. 4A).

In the PEECH trial, Feldman AM et al reported increased exercise time and peak O2.^[15]^ The study showed that after 6 months of intervention, 35% of participants in the EECP group and 25% of participants in the control group increased their exercise time by at least 60 s (*P* = .016). However, there was no significant statistical difference in peak VO2 changes between 2 groups (*P* > .05).

Two RCTs reported the MLHFQ.^[11,15]^ There was no significant statistical difference in changes of MLHFQ score between the EECP groups and the control groups after 3 months of follow-up(random effects model: WMD, −9.28; 95% CI, −19.30 to 0.75; *P* = .07; Figure 4B). However, the heterogeneity was considerable (*P* < .00001, *I*^2^ = 96%).

### 3.5 Secondary outcomes

Four studies reported LVEF.^[9,11,13,14]^ The meta-analysis results suggested that EECP significantly improved LVEF levels, and compared with the control groups, the difference was statistically significant (random-effects model: SMD, 0.64; 95% CI,0.29 to 1.00; *P* = .0004; Fig. 4C). However, there was high heterogeneity (*P* = .05; *I*^2^ = 62%). When we deleted individual studies, there was no substantially change in the pooled result. By subgroup analysis based on different follow-up time, the heterogeneity was significantly reduced (Fig. 4C).

Five RCTs included the results of N-terminal pro-brain natriuretic peptide. ^[9–12,14]^Pooled results showed that compared with the control groups, the NT-pro-BNP levels in the EECP groups was significantly reduced (random-effects model: SMD= −0.61; 95%CI, −1.20 to −0.01; *P* = .04; Fig. 4D). However, there was high heterogeneity (*P* < .00001; *I*^2^=89%). By omitting the study of Zhang et al, 2019, the heterogeneity further reduced to 0% with *P* = .61.

Two RCTs reported SAES associated with EECP.^[14,15]^ One study showed that no SAES occurred during the treatment of EECP.^[14]^ Another study reported 3 cases of EECP-related SAES, including 1 of worsening heart failure,1 of pulmonary embolism, and 1 of deep vein thrombosis (Table 1).

## 4 Discussion

Here, we evaluated the impact of EECP on patients with CHF through a systematic review and meta-analysis of the existing literature. Overall, the results of meta-analysis indicate that EECP can significantly improve exercise capability and LVEF of CHF patients, and reduce the level of NT-proBNP. However, EECP did not show a significant statistical difference in improving the QOL of patients with CHF.

The basic working principle of EECP is similar to that of intraaortic balloon counterpulsation, but it is noninvasive.^[18]^ EECP can increase the aortic diastolic pressure wave, improve myocardial blood supply and enhance myocardial contractility by mechanical assistance under the trigger of ECG. The external counterpulsation device can track the changes of cardiac cycle during counterpulsation, obtain the accurate time of aortic valve opening and closing, and realize the accurate setting of filling and discharging time. Sequential counterpulsation pressurizes the air sacs of the legs, thighs and buttocks from far to near, so that the collapse of the proximal great artery is later than that of the distal limb artery, which is conducive to more arterial blood flow back to the aorta and further increase the diastolic perfusion pressure of aortic root.^[19]^ At present, the standard treatment protocol of EECP is a total of 36 hours over 6 weeks (6 days per week) or a total of 35 hours over 7 weeks (5 days per week) (5). In our study, all included studies adopted a standard protocol of EECP to reduce the heterogeneity of treatment time and better evaluate the effect of EECP on CHF.

The patients with HF showed a significant decrease in exercise endurance, which is known to be a powerful prognostic indicator. The decreased exercise tolerance is associated with reduced QOL and increased mortality. At present, several methods have been proposed to clinically estimate or directly assess exercise capacity.^[20]^ The New York Heart Association(NYHA) functional class is a useful tool in clinical practice, which can be used to stratify a large number of patients with HF. Its main advantage is easy to use. However, its disadvantage is also obvious. It has a certain degree of subjectivity and can not be used for quantitative measurement of exercise capacity. The quantitative evaluation methods of exercise capacity include 6MWT and cardiopulmonary exercise test (CPX). Each has its advantages. Among them, CPX is the gold standard method to evaluate exercise capacity and cardiorespiratory fitness of patients with suspected heart disease and non heart disease.^[21–23]^ CPX is helpful to better understand the mechanism of impaired motor ability. However, compared with 6MWT, CPX is more time-consuming and expensive, and requires specialized equipment and personnel. Therefore, 6MWT is more widely used in clinical practice under the condition of limited resources.^[24]^ Studies demonstrated that 6MWT distance <300m is an independent prognostic marker of cardiovascular death in patients with reduced left ventricular ejection fraction.^[25]^ Moreover, the 6MWD is strongly correlated to peak VO2. Through this meta-analysis, we found that the increase of 6MWD in EECP groups was significantly longer than that in the control groups, suggesting that EECP can improve exercise capacity of CHF patients. But the heterogeneity was considerable (*I*^2^ = 95%). Although we conducted a subgroup analysis based on different follow-up time, the source of heterogeneity is still unclear. This heterogeneity may be due to clinical heterogeneity. The studies included not only the participants with reduced ejection fraction but also those with intermediate ejection fraction. In addition, participants may have different etiopathogenesis for CHF, such as ischemic cardiomyopathy (IHF), hypertensive heart disease, etc.

Only the PEECH trial used peak VO2 to measure exercise capacity in patients with HF.^[15]^ The study showed that after 6 months of follow-up, the proportion of patients achieving at least a 60-second increase in exercise duration was higher in the EECP group, but the proportion of peak VO2 improvement was similar between the 2 groups. However, subgroup analysis of this study showed that in participants aged 65 or over, the proportion of exercise duration and peak VO2 improvement were both significantly higher in EECP group compared with the control group at 6 months of follow-up.^[17]^ The possible reason was that more patients in the elderly group had heart failure due to IHF. However, the improvement of EECP on IHF had been confirmed by some studies.^[26,27]^ Of course, no other relevant RCTs have been retrieved, so more studies are needed to assess the impact of EECP on peak VO2 in CHF patients in the future.

Patients with CHF often experience a variety of physical and psychological complications, such as fatigue, dyspnea, edema, sleep difficulties, anxiety and depression.^[28]^ These symptoms restrict the patient's physical and social activities, leading to poor QOL. Poor QOL is associated with longer hospitalization time and mortality rates, as well as higher costs imposed on health systems, families, and patients.^[29,30]^ Therefore, ensuring a good QOL is very important for most heart failure patients. QOL is a multidimensional concept, which is influenced by economic and social factors, life satisfaction, and the severity and stage of heart failure. The MHLFQ is the most commonly used special tool to assess patients’QOLs.^[31,32]^ The lower the score, the higher the QOL. Meta-analysis showed that after 3 months of follow-up, the MHLFQ score of EECP group was lower than that of the control group, but there was no statistical difference. The PEECH trial also showed that there was no significant difference in changes of MLHFQ score between the EECP group and the control group after 6 months of follow-up. Similarly, changes in MLHFQ total score did not differ statistically between treatment groups in the 65-or-older subgroup of the PEECH trial at any time point. We conclude that the benefits of EECP focus on physical improvement rather than overall clinical recovery. Of course, due to the lack of more relevant research, such a conclusion still needs to be demonstrated.

Our meta-analysis further demonstrated that EECP could significantly reduce NT-proBNP levels compared with the control groups. NT-proBNP plays an important role in the diagnosis and treatment of CHF and is considered as an independent prognostic marker in CHF.^[33,34]^ In the subgroup analysis of the landmark Paradge-HF trial, compared with patients whose concentration of NT-proBNP remained above 1000 pg/mL, patients whose NT-proBNP fell below 1000 pg/mL at 1 month after randomization incurred 59% fewer deaths or admissions with HF. This trial further confirmed that the decrease of NT-proBNP levels in patients with HF was associated with lower hospitalization rate and cardiovascular mortality. Similar to NT-proBNP, LVEF is also an independent prognostic indicator of CHF, and can indirectly reflect exercise capacity of patients with HF. Moreover, our meta-analysis indicated that EECP increased LVEF. Another meta-analysis showed that compared with patients with persistently reduced LVEF, patients with improved LVEF had a significantly lower risk of all-cause mortality.^[35]^

Most RCTs in the included studies did not report SAES associated with the use of EECP. Li et al. showed no serious adverse reactions associated with EECP.^[14]^ Arthur et al. showed that the incidence of serious adverse reactions related to EECP was 4.23%, and which involved were worsening heart failure, pulmonary embolism and deep vein thrombosis.^[15]^ However, it should be noted that the total number of adverse events and serious adverse events were equal in the EECP group and the control group. Therefore, as recommended by relevant guidelines, the use of EECP in CHF is acceptable, but further high-quality RCTs are needed, especially long term observational studies.^[5]^

## 5 Limitations

Although our meta-analysis has shown that EECP was beneficial for patients with HF, there are some potential limitations of this analysis. First of all, according to the Cochrane Handbook,^[36]^ most RCTs did not fully perform allocation concealment, which may lead to increased heterogeneity. Of course, in the meta-analysis, we found some of other factors that led to considerable heterogeneity, such as different etiology of HF, different classification of HF, different observation time of main indicators, different control group, etc. Secondly, most of the included studies had a small sample size, and no long-term follow-up study was conducted on CHF patients treated by EECP. Thirdly, most studies reported positive results, which may lead to publication bias. Finally, most studies were published in Chinese journals, which reduced the extrapolation of results.

## 6 Conclusions

Despite its limitations, our meta-analysis still has clinical value. Our findings showed that EECP can improve exercise capacity and LVEF of CHF patients, and reduce the levels of NT-proBNP. However, the evidence that EECP improves the QOL in patients with CHF is still insufficient. In view of the heterogeneity of existing data, more and more well-designed RCTs are needed to confirm the current research results and to further study the effects of EECP in CHF patients.

## Author contributions

**Conceptualization:** Zhao-Feng Zhou, Chun-Yang Wu.

**Data curation:** Zhao-Feng Zhou, Da-jie Wang, Chun-Yang Wu.

**Formal analysis:** Zhao-Feng Zhou, Da-jie Wang, Xu-Mei Li.

**Investigation:** Xu-Mei Li, Cheng-Lin Zhang.

**Methodology:** Zhao-Feng Zhou, Xu-Mei Li, Cheng-Lin Zhang, Chun-Yang Wu.

**Project administration:** Cheng-Lin Zhang.

**Resources:** Xu-Mei Li, Cheng-Lin Zhang.

**Software:** Zhao-Feng Zhou, Cheng-Lin Zhang.

**Supervision:** Cheng-Lin Zhang.

**Validation:** Xu-Mei Li, Cheng-Lin Zhang, Chun-Yang Wu.

**Visualization:** Xu-Mei Li, Cheng-Lin Zhang.

**Writing – original draft:** Zhao-Feng Zhou, Da-jie Wang.

**Writing – review & editing:** Zhao-Feng Zhou, Chun-Yang Wu.
